# Delayed Diagnosis of a Giant Abdominal Aortic Aneurysm Complicating to Aortoenteric Fistula: A Case Report and Literature Review

**DOI:** 10.1002/ccr3.71912

**Published:** 2026-01-20

**Authors:** Ola Gamal Badawi Khalil, Mohammed Eltaieb Ali Mohammed, Salah Eldin Mohamed Elmustafa Hassan, Khalid Alshibli Dafalla Gasmalla, Rania Ahmed Elsiddig Hassan, Yousif Omer Elgaili Yousif

**Affiliations:** ^1^ Kassala Teaching Hospital Kassala Sudan; ^2^ Wad Madani Cardiac Centre Wad Madani Sudan; ^3^ Sudan Medical Specialization Board Khartoum Sudan; ^4^ Internal Medicine Department Gezira University Wad Madani Sudan; ^5^ Alzaim Alazhari University Khartoum Sudan

**Keywords:** abdominal aortic aneurysm, back pain, endovascular aneurysm repair, fever, post operative care

## Abstract

A 53‐year‐old man with chronic back pain and systemic symptoms was diagnosed with a massive abdominal aortic aneurysm (AAA) and aortoenteric fistula (AEF), which can cause severe gastrointestinal bleeding and potentially fatal side effects. In patients with chronic back pain and systemic symptoms, this case emphasizes the value of early imaging and a high level of clinical suspicion for AAA. AEF and rupture are two major consequences of a delayed diagnosis. Improving results in such complicated vascular presentations requires prompt management and careful postoperative surveillance. The case highlights the importance of early imaging and clinical suspicion for AAA, as delayed diagnosis can lead to complications like AEF and rupture.

## Key Clinical Message

- Atypical presentation with fever and chronic back pain led to delayed diagnosis.
- Graft infection with Gram‐negative organisms required combined surgical and prolonged targeted antibiotic therapy.
- Early vascular imaging and multidisciplinary care are critical to improving outcomes in complex AAA cases, particularly in resource‐limited settings.

## 1 Introduction

An Abdominal Aortic Aneurysm (AAA) is defined as a diameter of 3.0 cm or greater, typically two standard deviations above the mean diameter for men [1]. With 4500 fatalities annually, AAA ranks as the 14th most common cause of death in the US [2]. According to reports, AAA was responsible for 167,200 deaths and 3 million disability‐adjusted life years globally in 2017. However, because post‐mortem rates are so low, these figures are probably erroneous [3]. Caucasians are more likely to have aneurysms (AAA) than Black and Asians, possibly due to racial disparities in access to screening and treatment. Risk factors include coronary heart disease, peripheral artery disease, smoking, male sex, older age, hypertension, and a family history of aneurysms. AAA has a negative correlation with diabetes [4].

Endovascular repair of the thoracic and abdominal aorta has significantly improved management of aortic aneurysms and other aortic diseases, with annual EVAR surgeries increasing by 600% since FDA authorization of endograft devices [5]. EVAR of the abdominal aorta offers several benefits over open aneurysm repair, including improved perioperative survival and a lower 30‐day death rate (1.6%) compared to open surgery in a comprehensive evaluation of 1532 patients [6]. Direct measures to lessen AAA growth and the need for repair are not suggested. It is not advised to use beta blockers, such as propranolol, statins, doxycycline, angiotensin‐converting enzyme inhibitors or angiotensin receptor blockers, and roxithromycin (which is not available in the US) to lower the risk of AAA growth and rupture [7].

AAA can result in rupture, infection, thrombotic blockage of the branch vessel, aorto‐enteric fistula, aorto‐caval fistula, pseudoaneurysm, and compression of nearby tissues if treatment is not received [8].

This case study highlights the challenges faced by African men with AAA during treatment, emphasizing the importance of early identification and action to prevent complications and ensure optimal patient care.

## 2 Case History/Examination

A 53‐year‐old male, known to have type 2 Diabetes Mellitus (DM) for 3 years, has been well controlled. He is not hypertensive and doesn't smoke. The patient presented with sudden severe back pain in the lumbar area, dull in nature, aggravated by walking and relieved by rest. Further history revealed a prolonged history of intermittent high‐grade fever associated with rigors and sweating, not relieved by paracetamol tabs.

The patient appears ill, with an average body weight, normal heart rate, and temperature of 38.4°C. Cardiovascular, respiratory, and CNS examinations revealed no abnormalities. Abdominal examination showed normal contours, inverted umbilicus, visible pulsation, tenderness in the umbilical area, and no superficial masses. Auscultation showed normal bowel sounds and a bruit.

## 3 Methods (Investigations and Treatment)

Laboratory tests showed normal levels of hemoglobin, total white blood cell count, platelet count, erythrocyte sedimentation rate, C‐reactive protein, and renal function tests along with negative viral screening.

**FIGURE 1:**
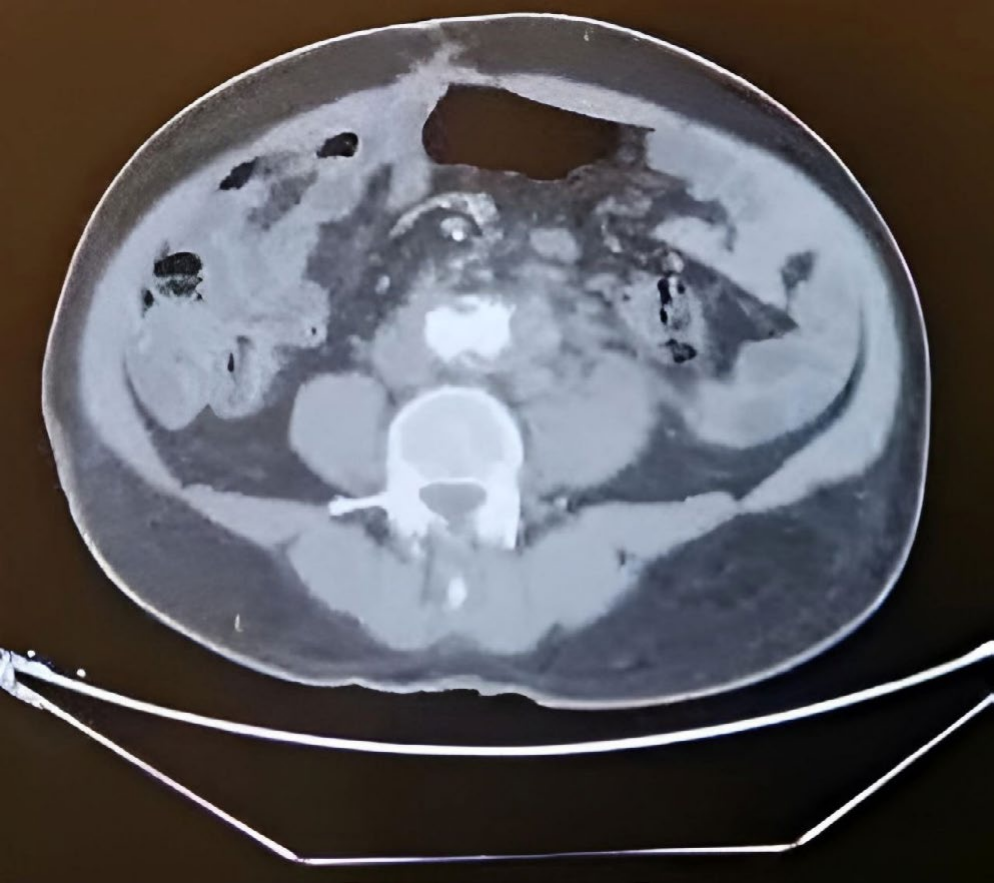
Axial contrast‐enhanced CT angiography (CTA) showing infrarenal abdominal aortic aneurysm with mural thrombus.

After that he was requested an Abdominal Ultrasound which showed large Abdominal aortic aneurysm (10.3 cm) with mural thrombus, no Renal stone or back pressure then the patient underwent CT angiography which revealed a long abdominal aorta aneurysm on the left side starting about 7 mm below the left renal artery, the maximum diameter is 12.3 mm with thrombosis, the true lumen is 5.9 cm, No iliac artery involvement and normal other organs as shown Figure 1.

### 3.1 First Surgery—Open Aneurysm Repair

The patient was referred to a consultant of cardiovascular and thoracic surgery and open aortic aneurysm repair was done, discharged in good condition.

### 3.2 Second Surgery—Endovascular Repair (EVAR)

**FIGURE 2:**
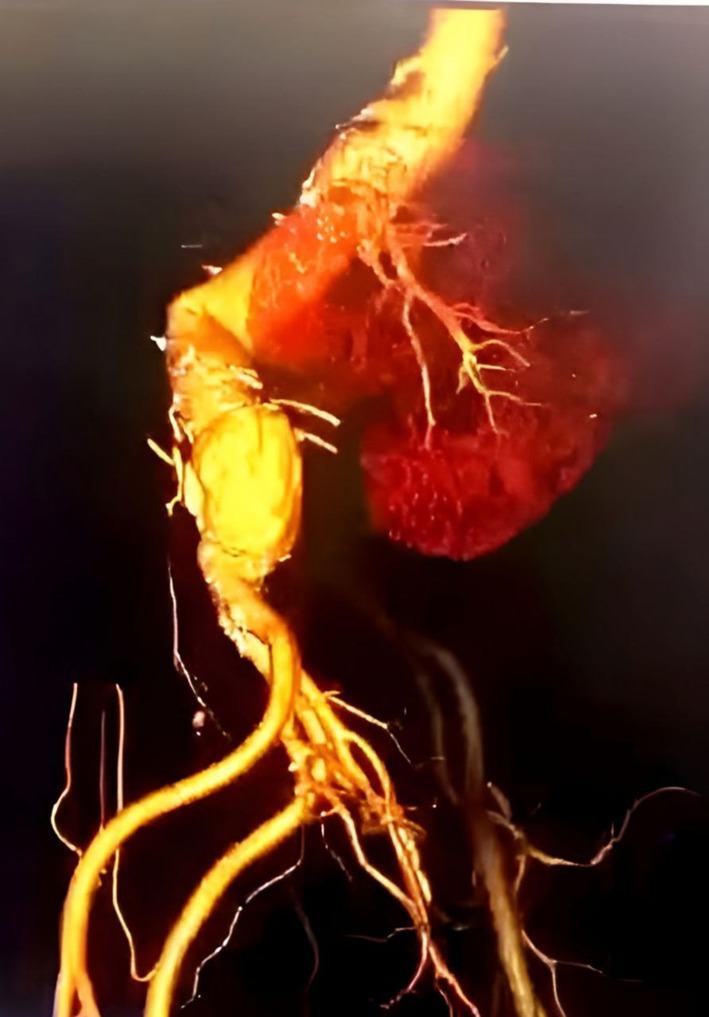
CT aortographic reconstruction showing ruptured infrarenal abdominal aortic aneurysm; including contrast extravasation and retroperitoneal hematoma.

**FIGURE 3:**
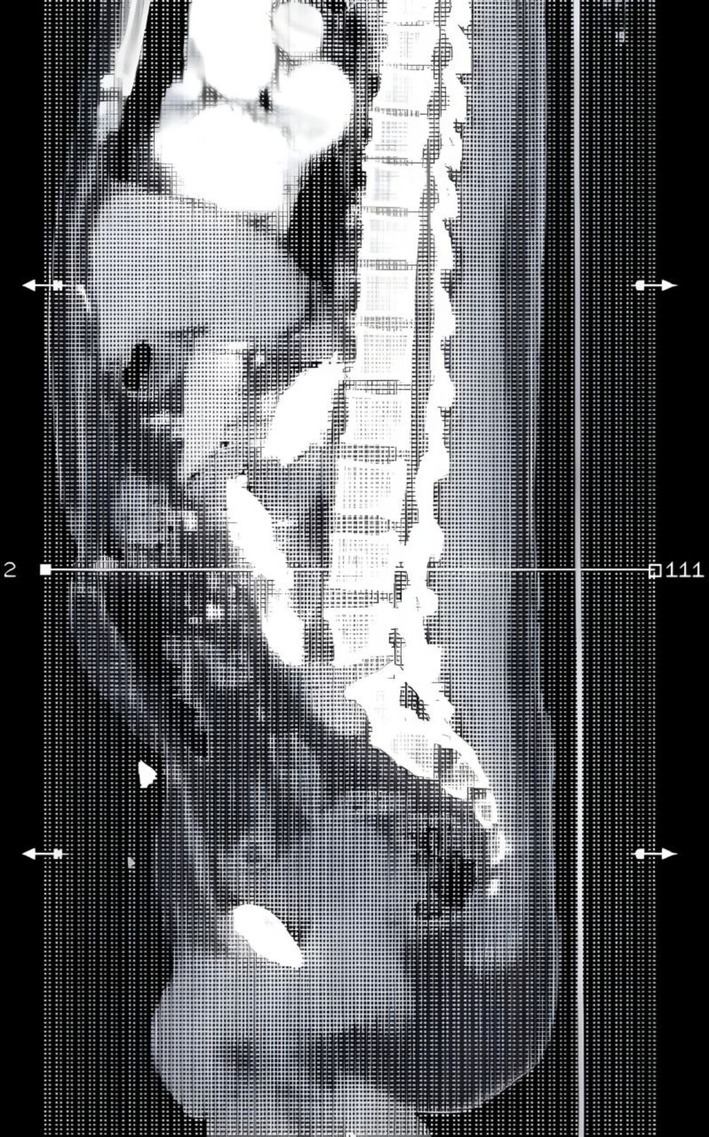
Sagittal reconstruction from a contrast‐enhanced computed tomography angiography (CTA) of the abdomen demonstrating a large infrarenal abdominal aortic aneurysm (AAA) with signs of rupture, evidenced by the disruption of the posterior aortic wall and retroperitoneal hematoma. The aorta is in close contact with adjacent bowel loops, raising suspicion for a secondary aorto‐enteric fistula. Loss of the fat plane between the aorta and bowel and possible air or contrast adjacent to the aneurysm suggest fistulous communication.

**FIGURE 4:**
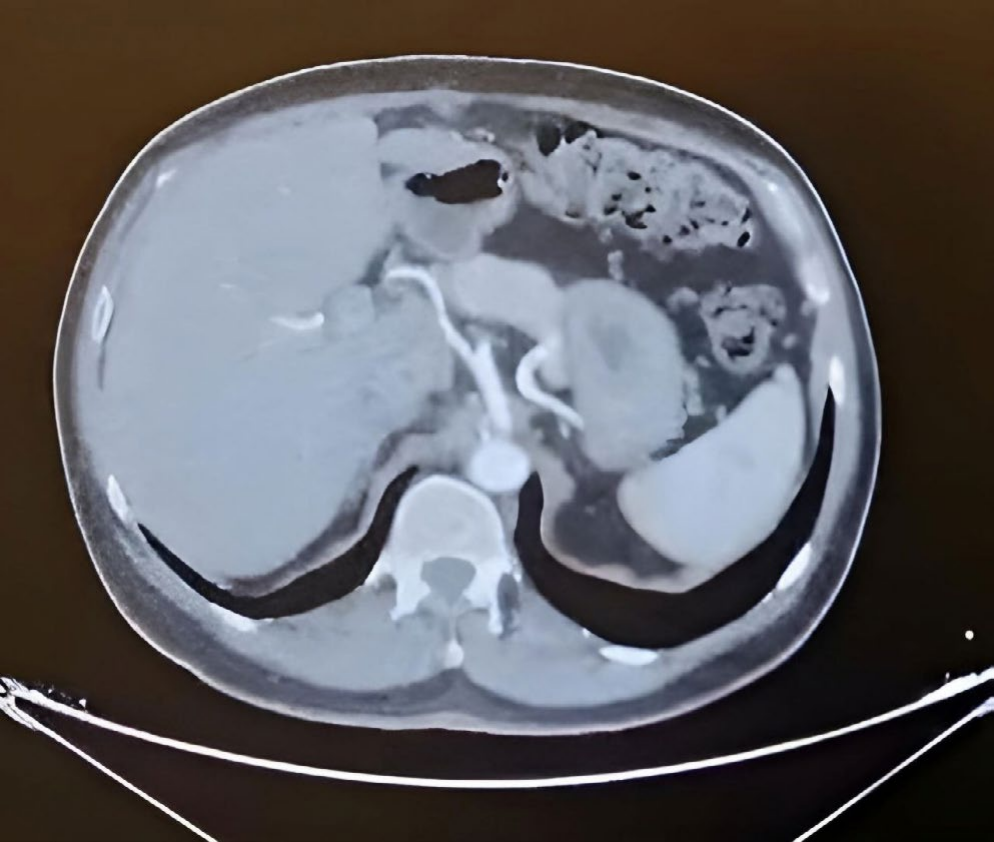
Axial contrast‐enhanced CT angiography (CTA) showing bifurcated endovascular stent graft after repair of infrarenal abdominal aortic aneurysm.

One month later the patient complained of vomiting blood that is large amount about one liter, associated with large clots and Malena, the patient received blood transfusion. Esophagogastroduodenoscopy (EGD) was normal, after stabilizing the patient repeated CT angiography showed 1.3 cm infrarenal large Abdominal aortic aneurysm 11.2 cm width with thrombosis up to 5 cm free aorta lumen, the aneurysm extends to the distal part of abdominal aorta, no iliac vessel involvement. Further, it showed signs of rupture (as shown in Figure 2) and evidence of aorto‐enteric fistula (AEF) as shown in Figure 3. The surgeon decided to go for endovascular aortic stent grafting (EVAR) to control the bleeding as shown in Figure 4.

After that the ENVR was done (as shown on Figure 3) and passed smoothly without complications; on postoperative day 1, the patient developed acute limb ischemia due to femoral artery embolism, and femoral embolectomy was done. Pt discharged with good condition on aspirin 75 mg, Rivaroxaban 2.5 mg tabs and proton pump inhibitors.

### 3.3 Third Surgery—Graft Explantation and In Situ Reconstruction

Several months later, he re‐presented with recurrent fever and abdominal pain. CT aortography showed graft infection with recurrent aorto‐enteric fistula. Bood and intraoperative cultures were positive for Gram‐negative organism ( *Enterobacter cloacae* ), and the patient received targeted intravenous antibiotic therapy for four weeks based on culture sensitivity, followed by surgical explantation of the infected graft and in situ aortic reconstruction using a prosthetic graft wrapped with omentum.

Postoperatively, he developed adhesive intestinal obstruction requiring re‐exploration and end‐to‐end bowel anastomosis. He later recovered and was discharged in stable condition under vascular and gastrointestinal follow‐up.

## 4 Conclusion and Results (Outcome and Follow‐Up)

The patient initially underwent open aortic aneurysm repair for a large infrarenal AAA and was discharged in stable condition. One month later, he presented with massive upper gastrointestinal bleeding due to an aorto‐enteric fistula, which was managed successfully with endovascular aortic repair (EVAR). He later developed recurrent symptoms with graft infection, for which positive cultures guided targeted intravenous antibiotic therapy, followed by graft explantation and in situ reconstruction using an omental wrap.

This case underscores the diagnostic challenge of atypical AAA presentations with fever and back pain and demonstrates the importance of a staged surgical approach (open repair → EVAR → in situ reconstruction) for infection control and hemodynamic stability. Early imaging and multidisciplinary management remain essential for improving outcomes in resource‐limited tropical settings.

## 5 Discussion

Acute abdominal aortic aneurysm (AAA) can be dangerous if not diagnosed promptly. Atypical symptoms like fever or back pain may be mistaken for viral or spinal disorders. This is illustrated in case reports like Kota et al.'s case of a diabetic man with intermittent fevers and back discomfort [9]. Similarly, Lourenço et al. described a 62‐year‐old man who had been experiencing back discomfort for three weeks without a fever, and whose workup unexpectedly showed a 20‐cm AAA [10]. Vascular imaging was delayed in both cases due to orthopedic or general reasons, resulting in 30% of patients having burst at presentation and 85% undergoing open repair of exceptionally large aneurysms, allowing aneurysm progression [11]. According to guidelines, imaging for AAA should be prompted in any patient with new back or abdominal discomfort and risk factors (age > 65, hypertension, smoking, and atherosclerotic disease) [7].

Anatomy and patient characteristics influence EVAR decision, with EVAR being less invasive and having lower perioperative mortality compared to open repair, with 30‐day mortality being around 1%–2% [12]. Postoperative monitoring is crucial for open repair patients, focusing on aneurysms and graft patency, while EVAR patients require lifelong imaging follow‐up for endoleaks. Overseeing massive AAAs is a significant task, with open repair often yielding stronger long‐lasting benefits [11, 12].

Our patient's presentation of fever and back pain has only been documented in a small number of comparable instances, according to a study of the literature. In addition to Kota et al.'s mycotic AAA, Van Wyngaarden et al. reported a patient who was referred to physical therapy for back discomfort but who, upon abdominal probing, had an unruptured AAA [9, 13]. Thus, this case aligns with prior reports in demonstrating that non‐specific symptoms of back pain and fever warrant early vascular imaging when initial workup is negative.

AAA repair can cause major issues like aortoenteric fistula and graft infection, which can be fatal. Diagnosis is challenging, and blood cultures may return negative. Common imaging indicators include CT or PET scans, with staph bacteria often found [14]. In a case study of nine post‐EVAR graft infections, all patients underwent explantation and either extra‐anatomic bypass or in situ grafting. Unfortunately, two patients died before discharge, and 33% had secondary aortoenteric fistulas, highlighting the need for aggressive intervention [15]. Similarly, Sumrein et al. report a case of an infected Dacron graft with lumbar discitis that was treated with antibiotics alone but ultimately resulted in the patient's death [14]. These reports illustrate that medical therapy alone is usually insufficient, surgical explantation or extensive debridement is often required for source control.

Another uncommon but deadly side effect of open AAA repair is an aortoenteric (aorto‐duodenal or aorto‐jejunal) fistula. It is estimated to occur in 0.4% to 1.6% of postoperative cases. When left untreated, it is nearly invariably fatal; even after surgery, 30%–40% of patients die [16]. Abdominal discomfort, gastrointestinal hemorrhage, or sepsis symptoms are possible presentations. Two of the three patients in the Laser series who had an aortoduodenal fistula and graft infection passed away during surgery [15].

Recent data from a five‐year Malaysian experience by Karthigesu et al. further emphasize that infective (formerly termed “mycotic”) aortic aneurysms in tropical regions can be caused by a range of organisms, particularly Salmonella spp. and *Burkholderia pseudomallei* , with infrarenal involvement being most common. Their study of 70 patients showed that both open and endovascular repairs can be effective when tailored to patient anatomy and resources, but long‐term antibiotic therapy and multidisciplinary care remain critical to outcomes [17]. In contrast to those primary infective native aneurysms, our case represents a secondary graft infection that developed as a postoperative complication following prior aneurysm repair, with positive cultures isolating a Gram‐negative organism ( *Enterobacter cloacae* ) that guided targeted intravenous antibiotic therapy. These findings collectively highlight that both regional microbiological patterns and the distinction between primary infective aneurysms and secondary postoperative graft infections play crucial roles in determining management strategies and patient outcomes, especially in tropical settings similar to Sudan.

Prompt recognition and aggressive treatment of aneurysms (AAA) are crucial for favorable outcomes. A larger study is recommended to determine the prevalence, risk factors, and outcomes of AAA, compare treatment options, and examine imaging modalities and management options. The article's conclusions may not be applicable to all AAA patients, as they are based on a single case report from Sudan and may not be applicable to other centers or nations.

## Author Contributions

**Ola Gamal Badawi Khalil:** conceptualization, project administration, resources, validation, visualization, writing – original draft, writing – review and editing. **Mohammed Eltaieb Ali Mohammed:** conceptualization, methodology, project administration, resources, validation, visualization, writing – original draft, writing – review and editing. **Salah Eldin Mohamed Elmustafa Hassan:** conceptualization, project administration, resources, validation, visualization, writing – original draft, writing – review and editing. **Khalid Alshibli Dafalla Gasmalla:** conceptualization, project administration, resources, validation, visualization, writing – original draft, writing – review and editing. **Rania Ahmed Elsiddig Hassan:** conceptualization, project administration, resources, validation, visualization, writing – original draft, writing – review and editing. **Yousif Omer Elgaili Yousif:** conceptualization, project administration, resources, validation, visualization, writing – original draft, writing – review and editing.

## Funding

The authors have nothing to report.

## Consent

Written informed consent was obtained from the patient to publish this report in accordance with the journal's patient consent policy.

## Conflicts of Interest

The authors declare no conflicts of interest.

## Data Availability

Data are available on request from the authors.
